# Sphingosine Prevents Rhinoviral Infections

**DOI:** 10.3390/ijms25052486

**Published:** 2024-02-20

**Authors:** Judith Lang, Matthias Soddemann, Michael J. Edwards, Gregory C. Wilson, Karl S. Lang, Erich Gulbins

**Affiliations:** 1 Department of Immunology, University Clinic, University of Duisburg-Essen, Hufelandstrasse 55, 45122 Essen, Germany; judith.lang@uk-essen.de (J.L.); karlsebastian.lang@uk-essen.de (K.S.L.); 2 Department of Molecular Biology, University Clinic, University of Duisburg-Essen, Hufelandstrasse 55, 45122 Essen, Germany; matthias.soddemann@uk-essen.de (M.S.); turninflat@icloud.com (M.J.E.); 3 Department of Surgery, College of Medicine, University of Cincinnati, Cincinnati, OH 45267, USA; wilsong3@ucmail.uc.edu

**Keywords:** sphingosine, rhinovirus, common cold, nose, infection, adverse effects

## Abstract

Rhinoviral infections cause approximately 50% of upper respiratory tract infections and novel treatment options are urgently required. We tested the effects of 10 μM to 20 μM sphingosine on the infection of cultured and freshly isolated human cells with minor and major group rhinovirus in vitro. We also performed in vivo studies on mice that were treated with an intranasal application of 10 μL of either a 10 μM or a 100 μM sphingosine prior and after infection with rhinovirus strains 1 and 2 and determined the infection of nasal epithelial cells in the presence or absence of sphingosine. Finally, we determined and characterized a direct binding of sphingosine to rhinovirus. Our data show that treating freshly isolated human nasal epithelial cells with sphingosine prevents infections with rhinovirus strains 2 (minor group) and 14 (major group). Nasal infection of mice with rhinovirus 1b and 2 is prevented by the intranasal application of sphingosine before or as long as 8 h after infection with rhinovirus. Nasal application of the same doses of sphingosine exerts no adverse effects on epithelial cells as determined by hemalaun and TUNEL stainings. The solvent, octylglucopyranoside, was without any effect in vitro and in vivo. Mechanistically, we demonstrate that the positively charged lipid sphingosine binds to negatively charged molecules in the virus, which seems to prevent the infection of epithelial cells. These findings indicate that exogenous sphingosine prevents infections with rhinoviruses, a finding that could be therapeutically exploited. In addition, we demonstrated that sphingosine has no obvious adverse effects on the nasal mucosa. Sphingosine prevents rhinoviral infections by a biophysical mode of action, suggesting that sphingosine could serve to prevent many viral infections of airways and epithelial cells in general. Future studies need to determine the molecular mechanisms of how sphingosine prevents rhinoviral infections and whether sphingosine also prevents infections with other viruses inducing respiratory tract infections. Furthermore, our studies do not provide detailed pharmacokinetics that are definitely required before the further development of sphingosine.

## 1. Introduction

Viral upper respiratory infections, so-called “common colds”, result in approximately 10 million patient appointments and exceed $22 billion in costs each year in the United States [1]. On average, common colds afflict adults two to three times each year. Children suffer substantially more infections than adults, for a total of more than one billion illnesses each year in the US. Much work has been directed toward the development of antiviral agents for preventing and treating upper respiratory infections. Unfortunately, the development of antiviral agents for upper respiratory tract infections has failed; currently there is no effective antiviral therapy for the common cold. The only treatment is to provide symptomatic relief while the illness runs its natural course.

Rhinoviruses are the main pathogens causing upper respiratory infections [2]. Although rhinoviral infections are usually not dangerous, they can become severe or even life threatening for patients with asthma, cystic fibrosis, or chronic obstructive pulmonary disease and for patients on a ventilator [3,4,5,6].

Rhinoviruses (RV) are small, positive-stranded RNA, non-enveloped viruses belonging to the picornavirus family. They infect human respiratory epithelial cells in the upper respiratory tract via intercellular adhesion molecule 1 (ICAM-1) or low-density lipoprotein 1 (LDL1) receptors, at least in vitro [7,8]. Most rhinoviruses initiate cellular infections by binding ICAM-1 and these rhinoviruses are categorized as major group rhinoviruses. Approximately 10% of rhinoviruses bind LDL1 receptors and are categorized as minor group rhinoviruses. A small third group binds to a presently unknown receptor [9]. However, it is unknown whether ICAM-1 and LDL1 receptors also mediate the infection in vivo, i.e., in the nasal tissue, with rhinoviruses.

Sphingolipids are localized in cellular membranes [10]. They determine biophysical membrane properties and are also involved in diverse cellular processes, including proliferation, cellular differentiation, apoptosis, signal transduction, and membrane trafficking [11,12,13,14]. Several studies have shown that acid sphingomyelinase, which converts sphingomyelin to ceramide at acidic pH values [15,16,17], plays an important role in bacterial infections and diseases caused by bacterial pathogens, such as bacterial pneumonia, meningitis, and sepsis [18,19]. In contrast to ceramide, which often promotes bacterial infections, sphingosine (2-amino-4-trans-octadecene-1,3-diol), which is released from ceramide by the activity of neutral, acid, or alkaline ceramidase [20,21,22], kills many bacterial pathogens and is an important component of the defense against bacterial pathogens on the skin and in the respiratory tract [23,24,25,26,27,28].

However, it is presently unknown whether sphingosine could also be exploited as an anti-viral compound.

The studies reported here examined whether extracellular sphingosine prevents rhinoviral infections of epithelial cells. We found that administering sphingosine intranasally to wildtype mice or treating human nasal epithelial cells with sphingosine prevents rhinoviral infections. Mechanistically, we found that sphingosine directly binds rhinoviruses, which seems to prevent infection. Thus, sphingosine may be a novel therapeutic drug for preventing the common cold.

## 2. Results

**Figure 1:**
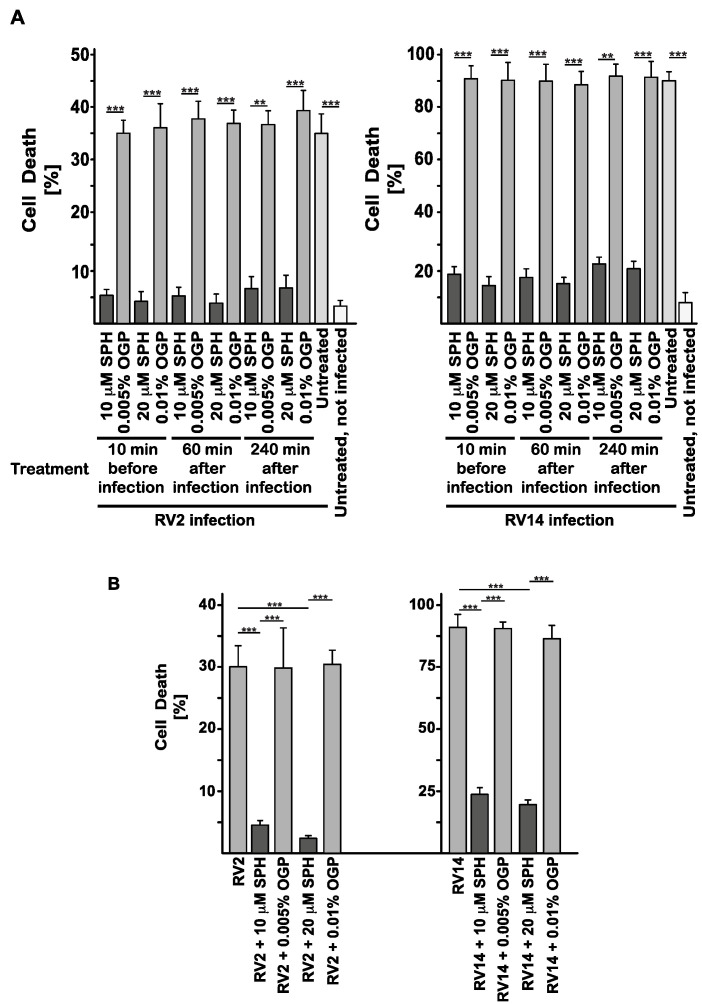
Sphingosine prevents the infection of human epithelial cells with rhinoviruses. (**A**) We infected 5 × 10^5^ HeLa cells for 4 days with 10^5^ plaque-forming units (PFU) each of rhinovirus strains RV2 (minor strain) or RV14 (major strain) in the presence or absence of 10 μM or 20 μM sphingosine (SPH), which was added either 10 min before infection with rhinoviruses or 60 min or 240 min after infection. Controls were either 0.005% or 0.01% octylglucopyranoside (OGP) alone, at concentrations corresponding to those used in the sphingosine samples, for cells that were infected but left untreated or cells that were left uninfected and untreated. Cell death caused by cytotoxicity of rhinoviruses was used as readout for the infection and was determined with Trypan Blue staining 4 days after the initiation of the infection. We determined the percentage of dead cells by counting 500 cells per sample. (**B**) We incubated 10^4^ PFU RV2 or RV14, respectively, with freshly isolated human nasal epithelial cells for 24 h in the presence of 10 μM or 20 μM sphingosine or the corresponding concentration of the solvent octylglucopyranoside. The cells were pelleted, supernatants were completely removed, and the cell pellets were resuspended in phosphate-buffered saline (PBS) and added to HeLa cells. Cells were incubated for 3 days, and cytotoxicity was determined as a measurement of viral titers. We determined the percentage of dead cells by counting 500 cells per sample. Shown are the means ± SD of the percentage of dead cells, *n* = 5 each in (**A**) and *n* = 4 each in (**B**). ** *p* < 0.01, *** *p* < 0.001, ANOVA.

To investigate the effects of sphingosine on rhinoviral infections, we added sphingosine to human epithelial HeLa cells at various concentrations before or after rhinoviral infection, measuring the cytopathic effects of rhinoviruses on HeLa cells. Sphingosine prevented infection with rhinovirus type 2 (RV2) and rhinovirus type 14 (RV14) at a concentration as low as 10 μM (Figure 1A). Increasing the sphingosine concentration to 20 μM did not significantly increase the inhibition of viral infections by sphingosine (Figure 1A). Sphingosine prevented the infection if it was added before the application of the virus or as long as 4 h after infection with the virus. Sphingosine itself had no effect on the cells. The solvent octylglucopyranoside, used as a control, did not alter the infection. We employed a bioassay, i.e., measuring the cytopathic effect of rhinoviruses on HeLa cells as a measurement for viral titers, because classical plaque-forming assays turned to be out very difficult and unreliable with rhinoviruses.

Next, we tested whether sphingosine also prevents the infection of freshly isolated human nasal epithelial cells with rhinovirus. The results showed that the infection of human nasal epithelial cells with 10^4^ plaque-forming units (PFU) of RV2 or RV14 was also abrogated in the presence of 10 μM or 20 μM sphingosine (Figure 1B).

**Figure 2:**
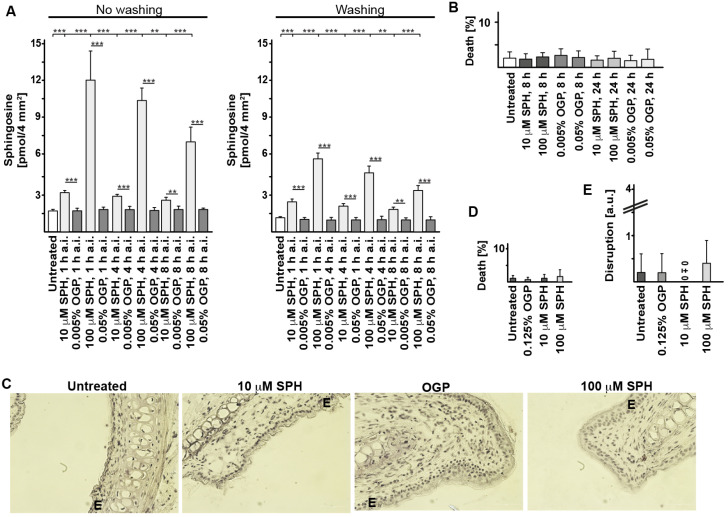
Nasal application of sphingosine in mice increases epithelial surface sphingosine concentrations and has no adverse effects. (**A**) Sphingosine (SPH) was applied into the nose of wildtype mice, and the concentration of sphingosine on the epithelial cell surface was determined after 1, 4 and 8 h. Sphingosine was applied at a volume of 10 μL in a 10 μM or a 100 μM sphingosine solution into the nostrils of wildtype mice. Control mice were given the solvent ocytylglucopyranoside (OGP) or were left untreated. Surface sphingosine concentrations were determined by an in situ kinase assay on the surface (2 mm × 2 mm) of the nasal epithelial cell layer. Washing the mucosa on the nasal bone before the kinase assay served to remove the mucus from the epithelial cell surface. Shown are the surface concentration of sphingosine, mean ± SD, *n* = 4. ** *p* < 0.01, *** *p* < 0.001, ANOVA. (**B**) Mice were treated 5 times, once every 12 h, with intranasally applied sphingosine at a concentration of 10 μM or 100 μM or with octylglucopyranoside at corresponding concentrations. Nasal tissue was removed 12 h after the last treatment and shock frozen, and frozen sections were stained for dead cells with terminal deoxynucleotidyl transferase dUTP nick end labeling (TUNEL) enzyme. We examined 100 cells each from 5 sections taken from each of 5 mice for dead cells; i.e., in total, we counted 2500 cells per group. Given is the mean ± SD of TUNEL-positive cells, *n* = 5. NOVA. (**C**–**E**) Mice were treated with sphingosine or octylglucopyranoside 5 times, once every 12 h. Nasal tissue was removed 12 h after the last treatment and shock frozen. Frozen sections were fixed and stained with hemalaun. (**C**) Displayed are typical results from 5 mice. The epithelial cell layer is indicated by an E. (**D**) Dead cells were identified by pycnotic or fragmented nuclei. We analyzed 100 cells each from 5 sections taken from each of 5 mice for dead cells (in total, we counted 2500 cells). (**E**) We analyzed the integrity of the epithelial cell layer after the application of sphingosine by using a scale ranging from 1 to 4, with 0 indicating a completely intact and with 4 indicating a disrupted epithelial cell layer. Shown are the mean ± SD of dead cells or the score of the epithelial cell layer integrity, *n* = 5. ANOVA.

To determine whether sphingosine also prevents rhinoviral infections in vivo, we first tested whether sphingosine could be administered intranasally to wildtype mice without adverse effects. Various concentrations of sphingosine were administered intranasally, and the concentration of sphingosine on the surface of the nasal mucosa was determined by an in situ kinase assay that we had previously developed to determine local surface concentrations of ceramide and sphingosine [15,17]. A local intranasal application of sphingosine resulted in a dose-dependent accumulation of sphingosine in the mucosa (Figure 2A). Washing the mucosa before the in situ kinase assay was performed and reduced the sphingosine accumulation in the nasal specimens on top of the epithelial cell layer, a finding indicating that most sphingosine remains in the mucus on top of the epithelial cell layer (Figure 2A).

Next, we determined whether the nasal application of sphingosine had any toxic adverse effects on nasal epithelial cells or on the integrity of the mucosa. The results showed that intranasally applied sphingosine neither induces cell death (Figure 2B–D) nor disrupts the nasal epithelial cell layer (Figure 2C,E).

**Figure 3:**
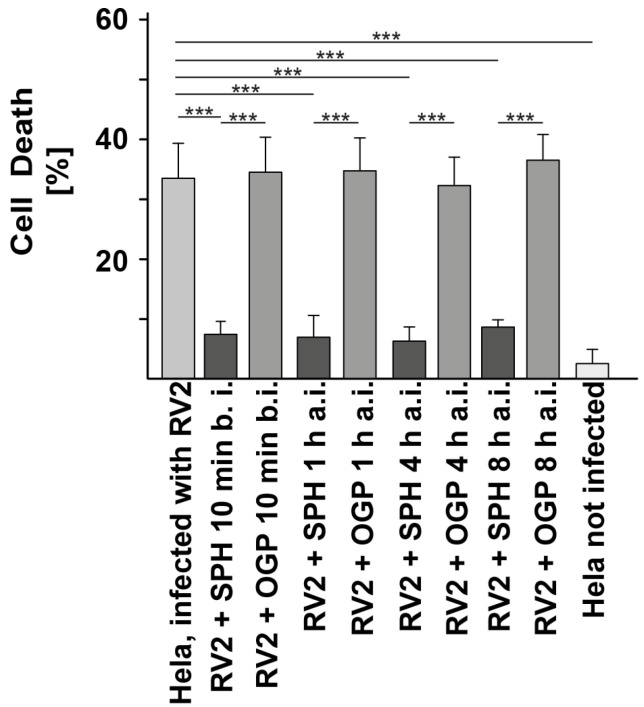
Sphingosine prevents infections with rhinovirus in vivo. Mice were intranasally infected with 10^3^ plaque-forming units (PFU) rhinovirus strain 2 (RV2) in 10 μL phosphate-buffered saline (PBS). Sphingosine (SPH) was applied into the nose of wildtype mice as a 100 μM suspension in PBS/octylglucopyranoside at a volume of 10 μL. Sphingosine was applied 10 min before rhinovirus infection (b.i.) and/or 1, 2, 3, 4, or 8 h after infection (a.i.). Controls received the solvent ocytylglucopyranoside (OGP) at the same concentration as that used when sphingosine was applied or were infected but left untreated or were left uninfected and untreated. Mice were sacrificed after 24 h. The mucosa was removed, cells were isolated and co-incubated with HeLA cells. Infection was analyzed by a bioassay measuring the cytopathic effect of the virus on HeLa cells after the release of the virus from nasal epithelial cells. The percentage of dead cells ± SD from 4 independent experiments is given. *** *p* < 0.001; ANOVA.

To determine whether sphingosine prevents rhinoviral infections in vivo, we infected wildtype mice by the intranasal application of RV2 and either treated the mice prophylactically with sphingosine before infection or applied sphingosine 1, 2, 3, 4, or 8 h after infection. Octylglucopyranoside administered at the same concentration served as a control. In these experiments, we removed the mucosa after infection, isolated single cells from the mucosa and incubated the nasal epithelial cells with HeLa cells. Virus that has been released from infected nasal epithelial cells infects HeLa cells and the infection in vivo can then be quantified by the cytopathic effect of Rhinovirus on HeLa cells. The results show that the application of sphingosine prevents nasal infections with RV2, even if it is administered as long as 8 h after infection (Figure 3).

**Figure 4:**
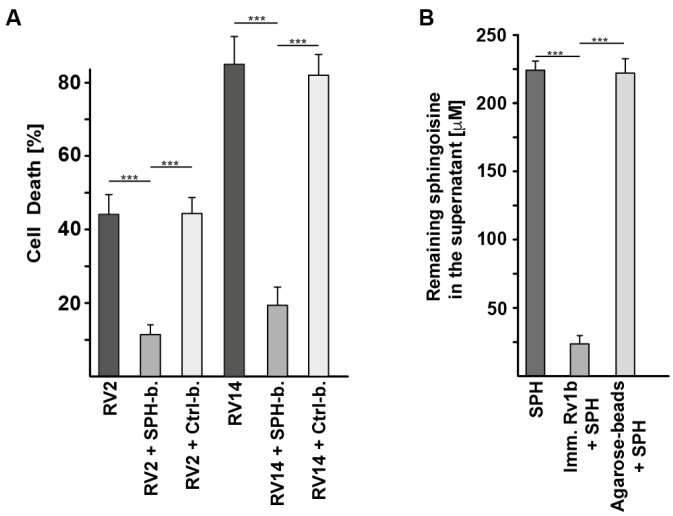
Sphingosine binds rhinovirus and neutralizes rhinovirus in nasal mucus by direct interaction via the NH_2_ group. (**A**) Sphingosine (SPH) that was immobilized to agarose beads (abbreviated SPH-b), or control beads (Ctrl-b) were incubated with 10^4^ plaque-forming units (PFU) of rhinovirus 2 or 14 (RV2 or RV14) for 4 h at 4 °C and pelleted by centrifugation. The complete supernatant was collected and incubated for 4 days with HeLa cells in 24-well plates for determination of the remaining viral titer in the supernatant. As readout, we determined the number of dead HeLa cells in aliquots of 500 cells after 4 days of culture. Given is the percentage of dead cells ± SD from 5 independent experiments each. *** *p* < 0.001, ANOVA. (**B**) RV1b was immobilized to agarose beads, and binding of sphingosine was measured. We incubated 1 μg/mL anti-RV1b antibodies with 10^6^ colony-forming units (CFU) RV1b for 1 h at 4 °C in H/S, immobilized the complex by adding agarose protein A/G beads, washed them extensively, and added 500 μL of a 250 μM sphingosine suspension in phosphate-buffered saline (PBS; pH 7.0). Samples were incubated for 4 h at 4 °C and pelleted. The supernatant was collected, and the amount of sphingosine remaining in the supernatants was determined by a kinase assay. Given is the mean ± SD of the remaining sphingosine concentration in the supernatant from 5 independent experiments each. *** *p* < 0.001, ANOVA.

Next, we elucidated the mechanisms by which sphingosine prevents infection with rhinovirus. To this end, we incubated RV2 and RV14 with sphingosine that was pre-bound to agarose beads or with control beads alone and determined the degree to which the virus was bound to sphingosine. The results showed that sphingosine-coupled agarose beads efficiently bind RV2 or RV14 and almost completely deplete the supernatant from any virus (Figure 4A). Similarly, we immobilized rhinovirus type 1b (RV1b) by immunoprecipitating it with a specific antibody and agarose beads and determined whether sphingosine bound to the virus. The results showed that the virus binds sphingosine with high effectiveness (Figure 4B). Controls that measured a potential release of sphingosine from the beads confirmed that sphingosine stably bound to the beads and the amount released from the beads in in the presence or absence of Rhinovirus was below the detection level of 10 pmol.

## 3. Discussion

The present study showed that sphingosine prevents infections of human nasal epithelial cells and in vivo nasal infections of mice with both minor and major group rhinoviruses. Mechanistically, we found that protonated sphingosine directly binds rhinoviruses in vitro and in vivo in the mucus on top of the epithelial cell layer. It is unknown how this interaction prevents infections with rhinoviruses. It might be possible that sphingosine alters the surface of the virus and thereby prevents infection and the initial contact of the virus with the epithelial cells. Lack of this initial contact prevents infection of these cells. This mechanism is in accordance with the notion that sphingosine can be applied prophylactically to prevent rhinoviral infections, because it will neutralize any virus infecting the nasal epithelium. It is also in accordance with the hypothesis that sphingosine even prevents further infection of nasal epithelial cells if it is applied after the initial infection, because it will prevent spreading of the virus to other epithelial cells, even though it does not revert the already existing infection. It is unknown how sphingosine binding may affect the virus. Further, the data do not exclude that sphingosine may target viral receptors on the mammalian/human cells, a mechanism that may contribute to prevention of the infection. Thus, future studies need to uncover the mechanism(s) how sphingosine alters viral infections.

We have previously shown that endogenous sphingosine plays an important role in infections with Herpes simplex virus 1 (HSV-1) [29]. Acid ceramidase, hydrolyzing ceramide to sphingosine, of macrophages traps herpes simplex virus in multivesicular bodies and protects from severe disease [29]. These studies revealed that infecting macrophages with HSV-1 results in uptake of the virus into endosomes, which mature to multivesicular bodies. Viral infections activate acid ceramidase, which generates sphingosine within intralumenal vesicles of these multivesicular bodies. Membrane-bound sphingosine traps the virus in the intralumenal vesicles and thereby prevents the release of the viral capsid into the cytoplasm. Instead, the virus is directed to lysosomes, where it is degraded. This endogenous defense mechanism might be mimicked in our studies by applying exogenous sphingosine, which may trap the virus in the mucus. In addition, if epithelial cells internalize the complex of exogenously applied sphingosine and virus, sphingosine will interact with the membrane of the endosome or the multivesicular body and will thereby trap the virus even within cells, resulting in a lysosomal degradation of the virus. In addition, it may be possible that some exogenous sphingosine binds to the plasma membrane and is even internalized by the epithelial cells, resulting in an increase in sphingosine concentrations at the infection site and in endosomes containing rhinovirus; this increased concentration of sphingosine will prevent further release of the virus into the cytoplasm and will promote intracellular degradation within lysosomes. It needs to be determined whether sphingosine also has a direct effect on molecules within the virus and whether such effects also contribute to the prevention of infection.

We have previously shown that infecting epithelial cells with rhinovirus results in an activation of acid sphingomyelinase and a release of ceramide, both of which are required for the uptake of rhinoviruses into the cells [30]. Thus, the virus exploits the acid sphingomyelinase-ceramide system for infection, but the host cell employs the acid ceramidase-sphingosine system to neutralize the virus. This suggests that the activity of the acid ceramidase might be a critical host factor determining infection susceptibility.

Our studies demonstrate that the binding of sphingosine to rhinovirus requires a neutral or slightly acidic pH, whereas alkalinization reduces or even abrogates the binding of sphingosine to the virus. This finding is consistent with a model in which the NH_2_ group in sphingosine is protonated and interacts with the negatively charged surface of the virus. The importance of the NH_2_ group for the antiviral effect of sphingosine is also consistent with previous findings showing that stearylamine also prevents viral infections [31,32,33].

Our studies demonstrate that the nasal application of sphingosine has no adverse effects on epithelial cells. We detected no cell death, influx of leukocytes, or disruption of the epithelial cell layer. This finding is consistent with recently published studies in which mini pigs inhaled sphingosine and exhibited no adverse effects [34]. This finding suggests that treatment with sphingosine is safe, although this suggestion certainly requires confirmation in further studies.

In summary, our findings indicate that the intranasal application of sphingosine prevents rhinoviral infections, at least in mice, and in human epithelial cells in vitro. Further preclinical and clinical studies are necessary for translating this finding to a treatment option for patients, but the present studies provide a clear path to develop a novel drug for the prevention and treatment of rhinoviral infections, and possibly also other viral infections of mucosal surfaces.

### Conclusions

Rhinoviral infections of the upper respiratory tract are among the most common infections worldwide. At present, no effective antiviral therapy for the common cold is available. Here, we demonstrate that the lipid sphingosine prevents infections with rhinovirus. We show that sphingosine binds, via its NH_2_ group, to the virus, traps the virus in the mucus on top of nasal epithelial cells and prevents the infection of murine and human nasal epithelial cells in vivo. The studies provide a novel paradigm of how lipids interfere with viral infections and provide a novel opportunity to prevent viral infections of the respiratory tract.

## 4. Materials and Methods

### 4.1. Ethical Statements

The experiments on human epithelial cells were approved by the local ethics committee of the University Hospital Essen, Germany, under the number 19-9033-BO, approved on the 10 December 2019.

All animal experiments were approved by the University of Cincinnati Ethic Committee and the Institutional Animal Care and Use Committee (IRB 10-05-10-01 from 8-6-2017 and IRB 2019–0324, revised under 20-07-07-01 on 3 December 2020). All experiments were performed according to the FELASA regulations and we also followed the ARRIVE guidelines.

### 4.2. Infection of Human Epithelial Cells

**Figure 5:**
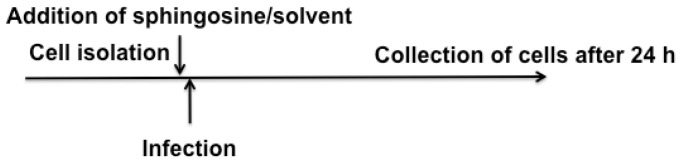
Experimental set up of the infection of human nasal epithelial cells.

Human nasal epithelial cells were obtained from 4 healthy volunteers by removing the cells from the nasal mucosa. To this end, a small brush was inserted into the nose (approximately 1.5 cm deep), gently rotated within the nose, and nasal epithelial cells were released from the brush and suspended immediately in 1 mL HEPES/Saline (H/S; 132 mM NaCl, 20 mM HEPES [pH 7.4], 5 mM KCl, 1 mM CaCl_2_, 0.7 mM MgCl_2_, 0.8 mM MgSO_4_). Nasal epithelial cells were pelleted by centrifugation at 2800 rpm for 8 min in an Eppendorf centrifuge (1710 g), resuspended in MEM supplemented with 10 mM HEPES (pH 7.4; Carl Roth GmbH, Karlsruhe, Germany), 2 mM L-glutamine, 1 mM sodium pyruvate, 100 μM nonessential amino acids, 100 U/mL penicillin, 100 μg/mL streptomycin and 1% fetal calf serum. Sphingosine was suspended as a 20 mM stock solution in an aqueous 10% octylglucopyranoside solution. Before its use, we sonicated the sphingosine stock in a bath-sonicator for 10 min to promote the formation of micelles, and we then diluted the sphingosine stock to the indicated concentration in 0.9% saline. We then added 10 μM or 20 μM sphingosine (final concentration) or the corresponding concentration of the solvent octylglucopyranoside followed by the addition of 10^4^ PFU RV2 or RV14 per sample freshly isolated human nasal epithelial cells. The infection was performed for 24 h at 33 °C in the presence of sphingosine or octylglucopyranoside (Figure 5). The cells were pelleted, supernatants were completely removed, and the cell pellets were resuspended in phosphate-buffered saline (PBS) and added to HeLa cells. Cells were incubated for 3 days and cytotoxicity on HeLa cells was determined by Trypan Blue staining. Cytotoxicity served as a measurement of viral titers. As classical plaque-forming assays turned out to be unreliable for rhinoviruses, this bioassay was used to determine viral titers.

### 4.3. Infection of HeLa Cells

HeLa cells were cultured in 24-well plates in Dulbecco’s modified Eagle medium (DMEM) supplemented with 2 mM L-glutamine, 1 mM sodium pyruvate, 100 μM nonessential amino acids, 100 U/mL penicillin, 100 μg/mL streptomycin (all from Invitrogen) and 10% fetal calf serum (FCS; PAA Laboratories GmbH, Coelbe, Germany). Cultures were grown until they reached approximately 70% density. Cells were washed twice in DMEM supplemented with 2% FCS and re-cultured in DMEM supplemented with 2% FCS. We then infected 5 × 10^5^ HeLa cells with 10^5^ PFU of either RV2 (minor strain) or RV14 (major strain) at 33 °C. Rhinovirus strains were obtained from ATCC, USA. Sphingosine (Avanti Polar Lipids, Alabaster, AL, USA) was added at a final concentration of 10 μM or 20 μM 10 min before infection with rhinoviruses or either 60 min or 240 min after infection with rhinoviruses. Sphingosine was diluted from a 20 mM stock in distilled water and 10% octylglucopyranoside (Sigma, Deisenhofen, Germany). Final octylglucopyranoside concentrations were 0.005% or 0.01% and these concentrations were used in the controls. Further controls were cells that were infected but not further treated or cells that were left untreated and uninfected. The samples were incubated for 4 h at 33 °C after the initiation of infection, the supernatant was removed, DMEM/10% FCS was added, and the cells were incubated for an additional 4 days. The supernatant was collected, the cells trypsinized and washed, the fractions combined and the cells stained with Trypan Blue (final concentration, 0.2%). Because rhinoviruses are cytotoxic, the number of dead cells accurately reflects the rhinoviral infection. We determined the percentage of dead cells by counting 500 cells per sample.

### 4.4. Mice and In Vivo Nasal Application of Sphingosine

We used C57BL/6 mice. Sphingosine was prepared as above. We applied 10 μL of either a 10 μM or a 100 μM sphingosine suspension into the nostrils of wildtype mice. The stock solution of sphingosine was diluted in 0.9% NaCl. Control mice were either left untreated or were treated with the solvent octylglucopyranoside at the same concentration as that used when sphingosine was applied. Mice were briefly anesthetized. For intranasal application, we used a blunt-ended 30G needle covered with a thin plastic film. The blunt-ended needle was inserted approximately 2 mm into the nostrils.

### 4.5. Surface Sphingosine Concentrations

Mice were sacrificed after 1, 4 or 8 h. The nasal bone was removed and placed on a 30 °C prewarmed plastic plate, and an area of 2 mm × 2 mm was immediately incubated with 0.001 units of sphingosine kinase 1 (#6068-SK-010; R&D Systems, Minneapolis, MN, USA) in 4 μL of 150 mM sodium acetate (pH 7.4), 1 μM adenosine triphosphate (ATP), and 10 μCi [^32^P]γATP (3000 Ci/mmol; Hartmann, Cologne, Germany) per sample. Control nasal tissues were incubated with the same buffer without sphingosine kinase or were left untreated. The sphingosine kinase reaction was performed for 15 min and terminated by adding the tissue into 100 μL H_2_O, followed by the addition of 20 μL 1N HCl, 800 μL CHCl_3_:CH_3_OH:1N HCl (100:200:1, *v*/*v*/*v*), and 240 μL each of CHCl_3_ and 2 M KCl. The lower phase was collected, dried, dissolved in 20 μL CHCl_3_:CH_3_OH (1:1, *v*/*v*), and separated on Silica G60 thin-layer chromatography (TLC) plates with CHCl_3_:CH_3_OH:acetic acid:H_2_O (90:90:15:5, *v*/*v*/*v*/*v*) as a developing solvent. The TLC plates were analyzed with a phosphorimager. Surface sphingosine levels were determined with a standard curve of C18-sphingosine.

We performed these assays without washing the mucosa on the nasal bone, i.e., immediately after removal, or after extensive washing of the specimen in PBS to remove mucus from the epithelial cell surface.

### 4.6. Toxicity Measurements after Nasal Application of Sphingosine

Sphingosine and octylglucopyranoside were applied into the nose of wildtype mice as described above. Mice were sacrificed and the nasal bone with the tissue was removed and incubated in Trypsin solution for 10 min for release and isolation of epithelial cells. Cells were washed twice in H/S (20 mM HEPES, 132 mM NaCl, 5 mM KCl, 1 mM CaCl_2_, 0.7 mM MgCl_2_, 0.8 mM MgSO_4_, pH 7.4), stained with Trypan Blue (0.2% final concentration), and dead cells were counted. We counted a total of at least 500 cells per sample.

In addition, we injected 10 μL of a 0.05% octylglucopyranoside, 10 μM sphingosine (in 0.005% octylglucopyranoside), or 100 μM sphingosine (in 0.05% octylglucopyranoside) suspension into the nose of each mouse 5 times, once every 12 h. Mice were sacrificed 12 h after the last application, nasal tissue was excised, and frozen sections were obtained. Sections were air dried for 5 min, fixed in ice-cold acetone for 5 min, and stained with hemalaun (#T865.2; Carl Roth, Karlsruhe, Germany). Furthermore, we quantified cell death by terminal deoxynucleotidyl transferase dUTP nick end labeling (TUNEL) staining. To this end, frozen sections of the nasal tissue were air dried for 5 min, fixed in ice-cold acetone for 5 min, and stained with TUNEL enzyme (Roche, Basel, Switzerland). The sections were treated in 0.1 M sodium citrate (pH 6.0) in a microwave at 450 W for 5 min and washed twice in PBS; the TUNEL reaction was performed with 5 μL TUNEL enzyme, 20 μL tetramethylrhodamine (TMR) labelling, and 25 μL TUNEL dilution buffer according to the vendor’s instructions. Samples were incubated for 60 min at 37 °C and washed 3 times in PBS. The samples were then incubated in PBS at 70 °C for 10 min to reduce background staining, washed once in PBS, and finally embedded in Mowiol (Sigma, Deisenhofen, Germany). Incubating the samples with DNAse served as a positive control for the TUNEL reaction. We then analyzed at least 500 cells in the nasal epithelial cell layer and determined the percentage of TUNEL-positive cells.

### 4.7. Hemalaun Stainings

Sections were stained with hemalaun (Carl Roth, Karlsruhe, Germany) for 5 min, washed, dehydrated in an alcohol series and Xylol, and embedded in Eukitt (#03989; Millipore, Darmstadt, Germany). Sections were analyzed by light microscopy. Hemalaun staining was performed to investigate whether the inhalation of sphingosine had any adverse effects on the morphology of the epithelial cell layer and whether sphingosine inhalation induced an influx of leukocytes. To this end, we used a score for analysis: Grade 0, no change in the epithelial cell layer, basal membrane intact, no evidence of leukocyte influx, less than 2% pyknotic (dead) epithelial cells; Grade 1, small disruptions in the epithelial cell layer, basal membrane intact, very minor leukocyte influx with few singular cells in the epithelial cell layer, less than 5% pycnotic epithelial cells; Grade 2, larger disruptions in the epithelial cell layer, basal membrane still intact, scattered leukocyte influx, less than 10% pyknotic epithelial cells; Grade 3, larger disruptions in the epithelial cell layer, disrupted basal membrane, massive leukocyte influx, more than 10% pycnotic epithelial cells. We analyzed 200 cells per specimen, 2 specimens per mouse, and 4 mice, for a total of 1600 epithelial cells per group.

### 4.8. In Vivo Infections

**Figure 6:**
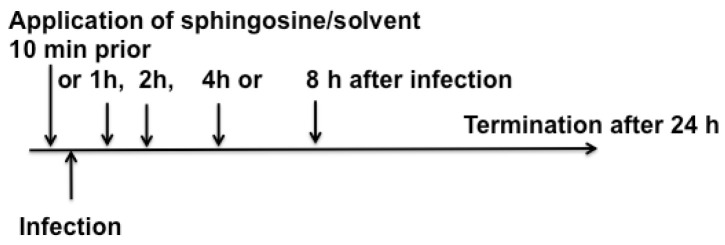
Experimental set up of the in vivo infection experiments.

Mice were infected with 10^3^ PFU RV2 in 10 μL PBS. The virus was applied directly into the nose with a blunt-ended, plastic-coated 30G needle, which was inserted approximately 2 mm into the nose. Sphingosine was applied into the nose of wildtype mice at a concentration of 100 μM in PBS at a volume of 10 μL. Sphingosine was diluted in 0.9% NaCl as described above. Sphingosine was applied either 10 min before rhinovirus infection or 1, 2, 3, 4 or 8 h after infection. Control animals were treated with the solvent, octylglucopyranoside, at the same concentration as that used when sphingosine was applied or were infected but left untreated. Mice were sacrificed after 24 h (Figure 6). The nasal bone with the nasal tissue was removed and incubated for 4 days with HeLa cells in a 24-well plate. If nasal epithelial cells are infected, rhinovirus is released because of the cytopathic effect of the virus, and the in vivo infection can then be determined and quantified by measuring the cell death of HeLa cells as a bioassay. Cell death was determined by Trypan Blue as described above by counting at least 500 cells per sample.

### 4.9. Sphingosine-Rhinovirus Binding

Sphingosine that was immobilized to agarose beads (Echelon Biosciences, Salt Lake City, UT, USA) or control beads alone were incubated with 10^4^ PFU RV2 or RV14 for 4 h at 4 °C at a volume of 400 μL PBS. The beads were then centrifuged for 2 min at 20,000× *g*, and the supernatant was completely collected. To determine the binding of the virus to sphingosine, and thereby the depletion of the virus from the supernatant, we quantified the remaining viral titer in the supernatant. To this end, we incubated the supernatants for 4 days with HeLa cells in 24-well plates and measured the number of dead cells as readout for remaining virus. We determined the number of dead HeLa cells by Trypan Blue staining and counting of at least 500 cells.

To immobilize RV1b to agarose beads, we incubated 1 μg/mL anti-RV1b antibodies (MyBiosource MBS 190867, San Diego, CA, USA) with 10^6^ PFU RV1b for 1 h at 4 °C in H/S, added 50 μL agarose protein A/G beads (Santa Cruz Inc., Heidelberg, Germany), incubated the samples for an additional 1 h at 4 °C, and washed the immobilized precipitates 5 times in H/S. Beads were then resuspended in 500 μL PBS (pH 7.0) containing 250 μM sphingosine. Samples were incubated for 4 h at 4 °C and pelleted, and aliquots of the supernatants were added to 50 mM HEPES (pH 7.4), 250 mM NaCl, 30 mM MgCl_2_, 0.001 units sphingosine kinase (R&D Systems, Minneapolis, MN, USA), 1 mM ATP, and 10 μCi [^32^P]γATP. The kinase reaction was performed for 1 h at 30 °C and was stopped by the addition of 20 μL 1N HCl, 800 μL CHCl_3_/CH_3_OH/1N HCl (100:200:1, *v*/*v*/*v*), 240 μL CHCl_3_, and 2 M KCl. Phases were separated; the lower phase was collected, dried, dissolved in 20 μL CHCl_3_:CH_3_OH (1:1, *v*/*v*), and separated on Silica G60 TLC plates with CHCl_3_/CH_3_OH/acetic acid/H_2_O (90:90:15:5, *v*/*v*/*v*/*v*). The TLC plates were exposed to radiography films, spots were removed from the plates, and the incorporation of [^32^P] into sphingosine was measured by liquid scintillation counting. Sphingosine concentrations were determined according to a standard curve of C18-sphingosine as above.

To determine the stability of sphingosine on the beads in the presence or absence of Rhinovirus, sphingosine beads were incubated with the virus or just buffer as above, centrifuged for 2 min at 20,000× *g* and sphingosine was quantified in the supernatants. To this end, 200 μL of the supernatants (the entire supernatant) were extracted in 600 μL CHCl_3_:CH_3_OH:1N HCl (100:100:1, *v*/*v*/*v*). Phases were separated by 5 min centrifugation, the lower phase was collected, dried in a Speedvac and resuspended in 20 μL of a detergent solution consisting of 7.5% [*w*/*v*] n-octyl glucopyranoside, 5 mM cardiolipin in 1 mM diethylenetriaminepentaacetic acid. Micelles were obtained by sonication of the samples in a Branson bath sonicator for 10 min. The kinase reaction was started by the addition of 80 μL of 0.001 units sphingosine kinase in 50 mM HEPES (pH 7.4), 250 mM NaCl, 30 mM MgCl_2_ 1 mM ATP and 10 μCi [^32^P]γATP and performed for 30 min at 30 °C under constant shaking. The reaction was terminated by extraction of the samples in 20 μL 1N HCl followed by addition of 800 μL CHCl_3_:CH_3_OH:1N HCl (100:200:1, *v*/*v*/*v*), and 240 μL each of CHCl_3_ and 2 M KCl. Samples were vortexed between additions. Phases were separated, the lower phase was collected, dried, dissolved in 20 μL CHCl_3_:CH_3_OH (1:1, *v*/*v*), and separated on Silica G60 TLC plates with CHCl_3_:CH_3_OH:acetic acid:H_2_O (90:90:15:5, *v*/*v*/*v*/*v*) as developing solvent. The TLC plates were analyzed with a phosphorimager. Sphingosine levels were determined with a standard curve of C18-sphingosine. The lower detection level of this assay is approximately 10 pmol sphingosine total.

### 4.10. Quantification and Statistical Analysis

Data are expressed as arithmetic means ± SD. To compare continuous variables from independent groups we used Student’s *t*-test for two groups and one-way ANOVA for more than two groups followed by post hoc Student’s *t*-tests for all pairwise comparisons, with Bonferroni corrections for multiple testing. The *p* values for the pairwise comparisons were calculated after Bonferroni correction. All values were normally distributed. The sample size planning was based on two-sided Wilcoxon–Mann–Whitney tests (G*Power, Version 3.1.7; Heinrich Heine University, Duesseldorf, Germany). Investigators were blinded to histologic analyses and animal identity.

## Data Availability

All data are included in the present manuscript.

## List of Abbreviations

a.i.	after infection
ATP	adenosine triphosphate
b.i.	before infection
FCS	fetal calf serum
H/S	HEPES-Saline buffer
HSV-1	Herpes simplex virus 1
ICAM-1	intercellular adhesion molecule 1
LDL1	low-density lipoprotein 1
OGP	octylglucopyranoside
PBS	phosphate-buffered saline
PFU	plaque-forming units
RNA	ribonucleic acid
RV	rhinovirus
SD	standard deviation
SPH	Sphingosine
TLC	thin-layer chromatography
TUNEL	Terminal deoxynucleotidyl transferase dUTP nick end labeling

## References

[1] Thomas M., Bomar P.A. (2019). Upper Respiratory Tract Infection.

[2] Schüz M.L., Dallmeyer L., Fragkou P.C., Omony J., Krumbein H., Hünerbein B.L., Skevaki C. (2023). Global prevalence of respiratory virus infections in adults and adolescents during the COVID-19 pandemic: A systematic review and meta-analysis. Int. J. Infect. Dis..

[3] Kumar N., Brar T., Kita H., Marks L.A., Miglani A., Marino M.J., Lal D. (2023). Viruses in chronic rhinosinusitis: A systematic review. Front. Allergy.

[4] Spector C., De Sanctis C.M., Panettieri R.A., Koziol-White C.J. (2023). Rhinovirus induces airway remodeling: What are the physiological consequences?. Respir. Res..

[5] Gonzalez-Rosales N., Kasi A.S., McCracken C.E., Silva G.L., Starks M., Stecenko A., Guglani L. (2023). Impact of viral respiratory infections on pulmonary exacerbations in children with cystic fibrosis. Pediatr. Pulmonol..

[6] Kiedrowski M.R., Bomberger J.M. (2018). Viral-bacterial co-infections in the cystic fibrosis respiratory tract. Front. Immunol..

[7] Greve J.M., Davis G., Meyer A.M., Forte C.P., Yost S.C., Marlor C.W., Kamarck M.E., McClelland A. (1989). The major human rhinovirus receptor is ICAM-1. Cell.

[8] Hofer F., Gruenberger M., Kowalski H., Machat H., Huettinger M., Kuechler E., Blass D. (1994). Members of the low density lipoprotein receptor family mediate cell entry of a minor-group common cold virus. Proc. Natl. Acad. Sci. USA.

[9] Bochkov Y.A., Watters K., Ashraf S., Griggs T.F., Devries M.K., Jackson D.J., Palmenberg A.C., Gern J.E. (2015). Cadherin-related family member 3, a childhood asthma susceptibility gene product, mediates rhinovirus C binding and replication. Proc. Natl. Acad. Sci. USA.

[10] Hannun Y.A., Obeid L.M. (2018). Sphingolipids and their metabolism in physiology and disease. Nat. Rev. Mol. Cell Biol..

[11] Dingjan T., Futerman A.H. (2021). The role of the ‘sphingoid motif’ in shaping the molecular interactions of sphingolipids in biomembranes. Biochim. Biophys. Acta Biomembr..

[12] Simons K., Ikonen E. (1997). Functional rafts in cell membranes. Nature.

[13] Stancevic B., Kolesnick R. (2010). Ceramide-rich platforms in transmembrane signaling. FEBS Lett..

[14] Huwiler A., Kolter T., Pfeilschifter J., Sandhoff K. (2000). Physiology and pathophysiology of sphingolipid metabolism and signaling. Biochim. Biophys. Acta.

[15] Quintern L.E., Schuchman E.H., Levran O., Suchi M., Ferlinz K., Reinke H., Sandhoff K., Desnick R.J. (1989). Isolation of cDNA clones encoding human acid sphingomyelinase: Occurrence of alternatively processed transcripts. EMBO J..

[16] Schuchman E.H., Suchi M., Takahashi T., Sandhoff K., Desnick R.J. (1991). Human acid sphingomyelinase. Isolation, nucleotide sequence and expression of the full-length and alternatively spliced cDNAs. J. Biol. Chem..

[17] Zeidan Y.H., Hannun Y.A. (2010). The acid sphingomyelinase/ceramide pathway: Biomedical significance and mechanisms of regulation. Curr. Mol. Med..

[18] Grassmé H., Jendrossek V., Riehle A., von Kurthy G., Berger J., Schwarz H., Weller M., Kolesnick R., Gulbins E. (2003). Host defense against Pseudomonas aeruginosa requires ceramide-rich membrane rafts. Nat. Med..

[19] Simonis A., Hebling S., Gulbins E., Schneider-Schaulies S., Schubert-Unkmeir A. (2014). Differential activation of acid sphingomyelinase and ceramide release determines invasiveness of Neisseria meningitidis into brain endothelial cells. PLoS Pathog..

[20] Coant N., Sakamoto W., Mao C., Hannun Y.A. (2017). Ceramidases, roles in sphingolipid metabolism and in health and disease. Adv. Biol. Regul..

[21] Bernardo K., Hurwitz R., Zenk T., Desnick R.J., Ferlinz K., Schuchman E.H., Sandhoff K. (1995). Purification, characterization, and biosynthesis of human acid ceramidase. J. Biol. Chem..

[22] Mao C., Obeid L.M. (2008). Ceramidases: Regulators of cellular responses mediated by ceramide, sphingosine, and sphingosine-1-phosphate. Biochim. Biophys. Acta.

[23] Bibel D.J., Aly R., Shinefield H.R. (1992). Antimicrobial activity of sphingosines. J. Investig. Dermatol..

[24] Fischer C.L., Walters K.S., Drake D.R., Blanchette D.R., Dawson D.V., Brogden K.A., Wertz P.W. (2013). Sphingoid bases are taken up by Escherichia coli and Staphylococcus aureus and induce ultrastructural damage. Ski. Pharmacol. Physiol..

[25] Pewzner-Jung Y., Tavakoli Tabazavareh S., Grassmé H., Becker K.A., Japtok L., Steinmann J., Joseph T., Lang S., Tuemmler B., Schuchman E.H. (2014). Sphingoid long chain bases prevent lung infection by Pseudomonas aeruginosa. EMBO Mol. Med..

[26] Grassmé H., Henry B., Ziobro R., Becker K.A., Riethmüller J., Gardner A., Seitz A.P., Steinmann J., Lang S., Ward C. (2017). β1-Integrin accumulates in cystic fibrosis luminal airway epithelial membranes and decreases sphingosine, promoting bacterial infections. Cell Host Microbe.

[27] Azuma M.M., Balani P., Boisvert H., Gil M., Egashira K., Yamaguchi T., Hasturk H., Duncan M., Kawai T., Movila A. (2018). Endogenous acid ceramidase protects epithelial cells from Porphyromonas gingivalis-induced inflammation in vitro. Biochem. Biophys. Res. Commun..

[28] Verhaegh R., Becker K.A., Edwards M.J., Gulbins E. (2020). Sphingosine kills bacteria by binding to cardiolipin. J. Biol. Chem..

[29] Lang J., Bohn P., Bhat H., Jastrow H., Walkenfort B., Cansiz F., Fink J., Bauer M., Olszewski D., Ramos-Nascimento A. (2020). Acid ceramidase of macrophages traps herpes simplex virus in multivesicular bodies and protects from severe disease. Nat. Commun..

[30] Grassmé H., Riehle A., Wilker B., Gulbins E. (2005). Rhinoviruses infect human epithelial cells via ceramide-enriched membrane platforms. J. Biol. Chem..

[31] Tahara K., Kobayashi M., Yoshida S., Onodera R., Inoue N., Takeuchi H. (2018). Effects of cationic liposomes with stearylamine against virus infection. Int. J. Pharm..

[32] Jeengar M.K., Kurakula M., Patil P., More A., Sistla R., Parashar D. (2021). Antiviral activity of stearylamine against chikungunya virus. Chem. Phys. Lipids.

[33] Jeengar M.K., Kurakula M., Patil P., More A., Sistla R., Parashar D. (2022). Effect of Cationic Lipid Nanoparticle Loaded siRNA with Stearylamine against Chikungunya Virus. Molecules.

[34] Carstens H., Schumacher F., Keitsch S., Kramer M., Kühn C., Sehl C., Soddemann M., Wilker B., Herrmann D., Swaidan A. (2019). Clinical development of sphingosine as anti-bacterial drug: Inhalation of sphingosine in mini pigs has no adverse side effects. Cell. Physiol. Biochem..

